# 73565 Defining tp53 tumor suppressor functions in zebrafish embryonal rhabdomyosarcoma

**DOI:** 10.1017/cts.2021.454

**Published:** 2021-03-30

**Authors:** Kunal Baxi, Jiangfei Chen, Amanda Lipsitt, Nicole Hensch, Long Wang, Abhik Bandopadhyay, Aaron Sugalski, Andrea Gilbert, Eleanor Chen, Peter Houghton, Gail Tomlinson, Myron Ignatius

**Affiliations:** University of Texas Health Science Center at San Antonio

## Abstract

ABSTRACT IMPACT: By assessing function of mutant (patient-specific) tp53 in zebrafish embryonal rhabdomyosarcoma will inform clinicians of the severity of mutant tp53 alleles. OBJECTIVES/GOALS: This study aims to define loss- and gain-of-function TP53 mutations by comparing effects in tp53-null and wild-type tumors. In addition, it aims to generate a rapid in vivo analysis platform to assign function to patient specific TP53 mutations in the clinic METHODS/STUDY POPULATION: To define tp53 function in ERMS pathogenesis, we previously generated a new tp53-null mutant (tp53-/-) in zebrafish by deleting the entire tp53 genomic locus using TALEN mutagenesis. tp53-/- zebrafish spontaneously develop a spectrum of tumors including sarcomas, leukemia and germ cell tumors (Ignatius…Baxi et. al., eLife) reminiscent of tumors observed in Trp53-null mice. Using the tp53-/- mutants to generate kRASG12D-induced ERMS, we discovered that tp53 is a potent repressor of metastases but rather surprisingly had no effect on self-renewal (Ignatius…Baxi et. al., eLife). Here, using tp53-/- zebrafish, we assessed effects of wild-type and mutant (patient specific) tp53 on tumor initiation, proliferation and apoptosis. RESULTS/ANTICIPATED RESULTS: ERMS tumor initiation in the tp53-/- background is observed in > 97% of animals whereas only <40% of wild-type animals develop ERMS. Additionally, tp53 is a potent suppressor of ERMS proliferation and its effect on apoptosis is minor. Next, we expressed either WT zebrafish or human TP53 in tp53-/- animals along with kRASG12D and both genes suppressed tumor initiation and growth. We co-expressed TP53C176F (found in two ERMS patients) and TP53P153del (identified in a patient with osteosarcoma in our clinic) in zebrafish ERMS, and find that the TP53C176F allele significantly suppressed tumor initiation with effects predominantly on enhanced apoptosis. However, the TP53P153del allele initiated tumors at similar frequency compared to tp53-/- animals but increased the initiation of tumors in the head musculature. DISCUSSION/SIGNIFICANCE OF FINDINGS: Different TP53 alleles identified in patient tumors have very different effects on tumorigenesis in vivo and can respond differently to potentially therapeutic compounds. Thus, the type of precision modeling demonstrated here promises to help further define patient-specific TP53 biology and improve clinical strategies in the future.

